# Crystal structure of bis­[*trans*-(ethane-1,2-di­amine-κ^2^ *N*,*N*′)bis­(thio­cyanato-κ*N*)chromium(III)] tetra­chlorido­zincate from synchrotron data

**DOI:** 10.1107/S2056989014027479

**Published:** 2015-01-01

**Authors:** Dohyun Moon, Jong-Ha Choi

**Affiliations:** a Pohang Accelerator Laboratory, POSTECH, Pohang 790-784, Republic of Korea; b Department of Chemistry, Andong National University, Andong 760-749, Republic of Korea

**Keywords:** Crystal structure, ethane-1,2-di­amine, thio­cyanate, *trans* isomer, chromium(III) complex, synchrotron data, hydrogen bonds

## Abstract

The structure of the title compound, [Cr(NCS)_2_(C_2_H_8_N_2_)_2_]_2_[ZnCl_4_], has been determined from synchrotron data. In the asymmetric unit, there are four independent halves of the Cr^III^ complex cations, each of which lies on an inversion centre, and one tetra­chlorido­zincate anion in a general position. The Cr^III^ atoms are coordinated by the four N atoms of two ethane-1,2-di­amine (en) ligands in the equatorial plane and two N-bound NCS^−^ anions in a *trans* arrangement, displaying a slightly distorted octa­hedral geometry with crystallographic inversion symmetry. The Cr—N(en) and Cr—N(NCS) bond lengths range from 2.0653 (10) to 2.0837 (10) Å and from 1.9811 (10) to 1.9890 (10) Å, respectively. The five-membered metalla-rings are in stable *gauche* conformations. The [ZnCl_4_]^2−^ anion has a distorted tetra­hedral geometry. The crystal structure is stabilized by inter­molecular hydrogen bonds involving the en NH_2_ or CH_2_ groups as donors and chloride ligands of the anion and S atoms of NCS^−^ ligands as acceptors.

## Abstract 2

The Cr^III^ atoms in the title compound show a distorted octa­hedral coordination with four N atoms of two ethane-1,2-di­amine ligands (en) in the equatorial plane and two N-coordinated NCS^−^ groups in axial positions. The [ZnCl_4_]^2−^ anion has a slightly distorted tetra­hedral geometry.

## Chemical context   

The study of geometrical isomers in octa­hedral transition metal complexes with bidentate amines has been an area of intense activity and has provided much basic structural information and insights into their spectroscopic properties. Ethane-1,2-di­amine (en) can act as a bidentate ligand to a central metal ion through its two nitro­gen atoms, forming a five-membered ring. The [Cr(en)_2_ *L*_2_]^+^ (where *L* is a monodentate ligand) cation can form either *trans* or *cis* geometric isomers. Infrared, electronic absorption and emission spectral properties are useful in determining the geometric isomers of chromium(III) complexes with mixed ligands (Choi, 2000*a* ▸,*b* ▸; Choi *et al.*, 2002 ▸, 2004*a* ▸,*b* ▸; Choi & Moon, 2014 ▸). However, it should be noted that the geometric assignments based on spectroscopic studies are much less conclusive. In addition, NCS^−^ is an ambidentate ligand because it can coordinate to a transition metal through the nitro­gen (*M*—NCS), or the sulfur (*M*—SCN), or both (*M–*-NCS—*M*). In general, hard metals such as chromium, nickel and cobalt tend to form metal–NCS bonds, whereas the soft metals such as mercury, rhodium, iridium, palladium and platinum tend to bind through the S atom. The oxidation state of the metal, the nature of other ligands and steric factors also influence the mode of coordin­ation.

Here, we report on the synthesis and structure of [Cr(en)_2_(NCS)_2_]_2_[ZnCl_4_] in order to determine the bonding mode of the thio­cyanate group and the geometric features of the two en ligands, the two NCS groups and the [ZnCl_4_]^2−^ anion. 
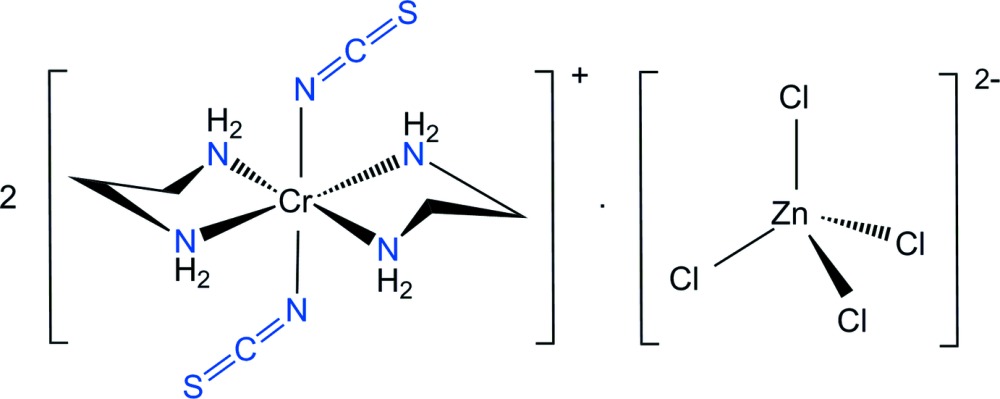


## Structural commentary   

**Figure 1:**
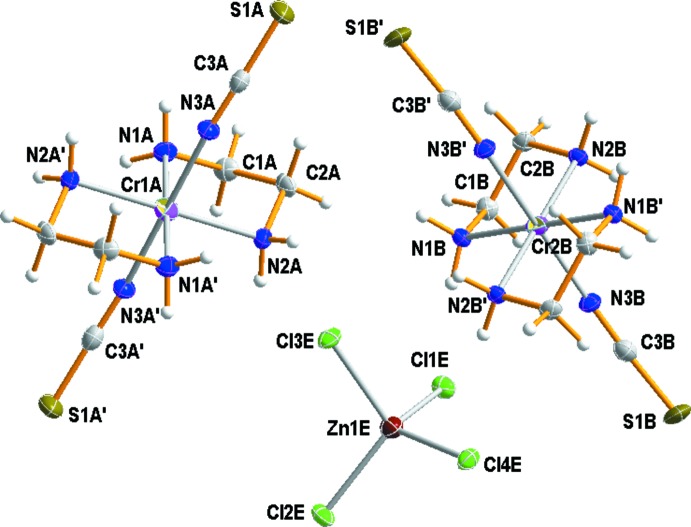
A perspective view (60% probability ellipsoids) of two independent chromium(III) complex cations and the unique tetra­chlorido­zincate anion in *trans*-[Cr(en)_2_(NCS)_2_]_2_[ZnCl_4_]. The symmetry code for A′ atoms is −*x* + 2, −*y*, −*z* + 1 and for B′ atoms, the symmetry code is −*x* + 1, −*y* + 1, −*z* + 1.

Structural analysis shows that there are four crystallographically independent Cr^III^ complex cations in which the four nitro­gen atoms of the two en ligands occupy the equatorial sites and the two thio­cyanate anions coordinate to the Cr^III^ atom through their N atoms in a *trans* configuration. Fig. 1 ▸ shows an ellipsoid plot of two independent complex cations and one anion in *trans*-[Cr(en)_2_(NCS)_2_]_2_[ZnCl_4_], with the atom-numbering scheme.

The asymmetric unit contains four halves of the [Cr(en)_2_(NCS)_2_]^+^ complex cations and one [ZnCl_4_]^2−^ anion. The four Cr^III^ atoms are located on crystallographic centers of symmetry, so these complex cations have mol­ecular *C_i_* symmetry. The spatial configuration of the bidentate en ring is a stable *gauche* form, which has been observed in other compounds (Brencic & Leban, 1981 ▸; Choi *et al.*, 2010 ▸). The carbon atoms in the en ring are arranged symmetrically above and below the plane defined by the chromium and the en nitro­gen atoms. The two Cr–en rings are in δ and λ conformations as the Cr^III^ atom occupies a special position with inversion symmetry. The Cr—N bond lengths for the en ligand range from 2.0653 (10) to 2.0837 (10) Å, in good agreement with those observed in *trans*-[Cr(en)_2_F_2_]ClO_4_ (Brencic & Leban, 1981 ▸), *trans*-[Cr(en)_2_Br_2_]ClO_4_ (Choi *et al.*, 2010 ▸), *trans*-[Cr(Me_2_tn)_2_Cl_2_]_2_ZnCl_4_ (Me_2_tn = 2,2-di­methyl­propane-1,3-di­amine) (Choi *et al.*, 2011 ▸) and *trans*-[Cr(2,2,3-tet)F_2_]ClO_4_ (2,2,3-tet = 1,4,7,11-tetra­aza­undeca­ne) (Choi & Moon, 2014 ▸). The Cr—N(CS) distances lie in the range 1.9811 (10) to 1.9890 (10) Å and are similar to the average values of 1.9826 (15) and 1.996 (15) Å found in *trans*-[Cr(Me_2_tn)_2_(NCS)_2_]NCS (Choi & Lee, 2009 ▸) and *cis*-[Cr(cyclam)(NCS)_2_]NCS (cyclam = 1,4,8,11-tetra­aza­cyclo­tetra­deca­ne) (Moon *et al.*, 2013 ▸), respectively. The N-coord­in­ating ­thio­cyanato groups are almost linear with N—C—S angles ranging from 177.11 (8) to 179.15 (9)°. The [ZnCl_4_]^2−^ counter-anion has a distorted tetra­hedral geometry due to the influence of hydrogen bonding on the Zn—Cl bond lengths and the Cl—Zn—Cl angles. Zn—Cl bond lengths range from 2.2518 (8) to 2.2923 (8) Å and the Cl—Zn—Cl angles are in the range 106.71 (2)–112.49 (2)°.

## Supra­molecular features   

**Figure 2:**
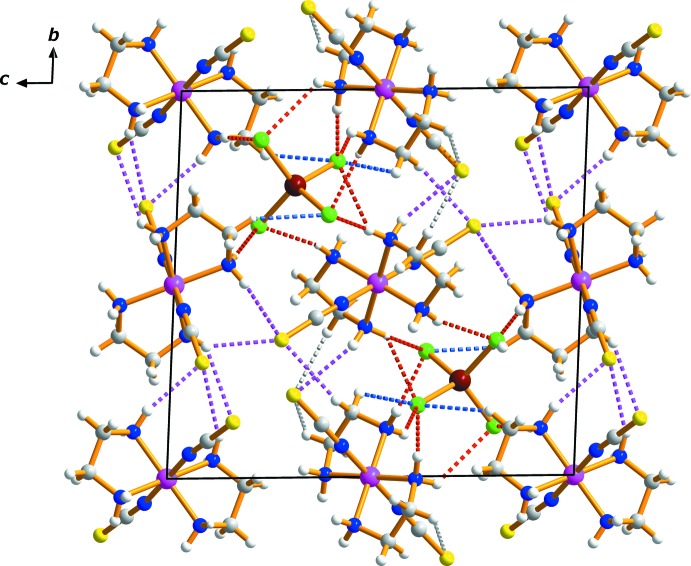
The mol­ecular packing for *trans*-[Cr(en)_2_(NCS)_2_]_2_[ZnCl_4_], viewed along the *a* axis. Hydrogen bonding is denoted by dashed liness, N—H⋯S (purple), C—H⋯S (grey), N—H⋯Cl (red), and C—H⋯Cl (blue).

In the asymmetric unit, a series of N—H⋯Cl and C—H⋯Cl hydrogen bonds link each anion to the four neighbouring cations, while N—H⋯S and C—H⋯S contacts inter­connect the complex cations (Fig. 2 ▸, Table 1 ▸). An extensive array of additional, similar contacts generate a three-dimensional network of mol­ecules stacked along the *a*-axis direction.

## Database survey   

A search of the Cambridge Structural Database (Version 5.35, May 2014 with one update; Groom & Allen, 2014 ▸) indicates a total of 13 hits for Cr^III^ complexes with a [Cr(en)_2_ *L*_2_]^+^ unit. The crystal structures of *trans*-[Cr(en)_2_Cl_2_]Cl·HCl·2H_2_O (Ooi *et al.*, 1960 ▸), *trans*-[Cr(en)_2_F_2_]*X* (*X* = ClO_4_, Cl, Br) (Brencic & Leban, 1981 ▸), *cis*-[Cr(en)_2_F_2_]ClO_4_ (Brencic *et al.*, 1987 ▸), *trans*-[Cr(en)_2_Br_2_]ClO_4_ (Choi *et al.*, 2010 ▸) have been reported previously. However, no structures of salts of [Cr(en)_2_(NCS)_2_]^+^ with any anions were found.

## Synthesis and crystallization   

All chemicals were reagent-grade materials and were used without further purification. The starting material, *trans*-[Cr(en)_2_(NCS)_2_]ClO_4_ was prepared according to the literature (Sandrini *et al.*, 1978 ▸). The crude perchlorate salt (0.10 g) was dissolved in 5 mL of 0.1 *M* HCl at 333 K and added to 2 mL of 6 *M* HCl containing 0.3 g of solid ZnCl_2_. The resulting solution was filtered and allowed to stand at room temperature for two days to give red crystals of the tetra­chlorido­zincate salt suitable for X-ray structural analysis.

## Refinement   

Crystal data, data collection and structure refinement details are summarized in Table 2 ▸. Hydrogen atoms bound to carbon or nitro­gen were placed in calculated positions (C—H = 0.95, N—H = 0.91 Å), and were included in the refinement using the riding-model approximation with *U*_iso_(H) set to 1.2*U*_eq_(C, N).

## Supplementary Material

Crystal structure: contains datablock(s) I. DOI: 10.1107/S2056989014027479/sj5433sup1.cif

Structure factors: contains datablock(s) I. DOI: 10.1107/S2056989014027479/sj5433Isup2.hkl

CCDC reference: 1039747

Additional supporting information:  crystallographic information; 3D view; checkCIF report

## Figures and Tables

**Table 1:** Hydrogen-bond geometry (, )

*D*H*A*	*D*H	H*A*	*D* *A*	*D*H*A*
N1*A*H1*A*1Cl3*E* ^i^	0.91	2.48	3.3700(13)	165
N2*A*H2*A*1Cl1*E* ^ii^	0.91	2.50	3.3483(13)	155
N2*A*H2*A*2Cl3*E*	0.91	2.90	3.5797(12)	133
C1*A*H1*A*3S1*A* ^iii^	0.99	2.91	3.5983(15)	127
C2*A*H2*A*3Cl3*E*	0.99	2.91	3.5533(15)	123
C2*A*H2*A*4S1*B* ^iv^	0.99	2.94	3.6270(15)	128
N1*B*H1*B*1Cl1*E*	0.91	2.45	3.2813(13)	152
N1*B*H1*B*2S1*A* ^iii^	0.91	2.81	3.5401(13)	138
N2*B*H2*B*1Cl4*E* ^iv^	0.91	2.49	3.3532(13)	159
N2*B*H2*B*2Cl1*E* ^v^	0.91	2.77	3.4934(12)	138
C1*B*H1*B*3S1*A* ^iii^	0.99	2.98	3.5910(14)	121
C1*B*H1*B*3S1*B* ^v^	0.99	2.87	3.6440(14)	136
C2*B*H2*B*3Cl1*E* ^v^	0.99	2.93	3.5309(14)	120
N1*C*H1*C*1Cl4*E*	0.91	2.40	3.3058(12)	171
N1*C*H1*C*2S1*B*	0.91	2.73	3.4473(14)	137
N2*C*H2*C*1S1*C* ^iii^	0.91	2.50	3.2836(12)	144
N2*C*H2*C*2S1*B* ^vi^	0.91	2.75	3.4063(11)	130
N2*C*H2*C*2S1*D* ^iii^	0.91	2.88	3.5893(13)	135
C1*C*H1*C*4Cl1*E*	0.99	2.86	3.7421(13)	149
N1*D*H1*D*1S1*C*	0.91	2.61	3.4937(13)	164
N1*D*H1*D*2Cl2*E*	0.91	2.49	3.3919(12)	172
N2*D*H2*D*1S1*C* ^vii^	0.91	2.78	3.6225(12)	155
N2*D*H2*D*2S1*D* ^viii^	0.91	2.67	3.3564(12)	133
C1*D*H1*D*3Cl3*E*	0.99	2.88	3.7357(14)	145
C1*D*H1*D*4Cl2*E* ^ii^	0.99	2.98	3.7397(12)	135

Symmetry codes: (i) 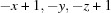; (ii) 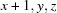; (iii) 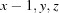; (iv) 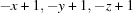; (v) 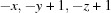; (vi) 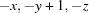; (vii) 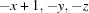; (viii) 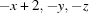.

**Table 2:** Experimental details

Crystal data	
Chemical formula	[Cr(NCS)_2_(C_2_H_8_N_2_)_2_]_2_[ZnCl_4_]
*M*_r_	783.90
Crystal system, space group	Triclinic, *P* 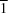
Temperature (K)	100
*a*, *b*, *c* ()	7.6870(15), 13.853(3), 14.560(3)
, , ()	92.74(3), 92.76(3), 90.21(3)
*V* (^3^)	1546.9(5)
*Z*	2
Radiation type	Synchrotron, = 0.62998
(mm^1^)	1.50
Crystal size (mm)	0.10 0.03 0.03

Data collection	
Diffractometer	ADSC Q210 CCD area detector
Absorption correction	Empirical (using intensity measurements) (*HKL3000sm *SCALEPACK**; Otwinowski Minor, 1997 ▸)
*T*_min_, *T*_max_	0.865, 0.956
No. of measured, independent and observed [*I* > 2(*I*)] reflections	17036, 8546, 8434
*R*_int_	0.014
(sin /)_max_ (^1^)	0.696

Refinement	
*R*[*F* ^2^ > 2(*F* ^2^)], *wR*(*F* ^2^), *S*	0.018, 0.049, 1.03
No. of reflections	8546
No. of parameters	322
H-atom treatment	H-atom parameters constrained
_max_, _min_ (e ^3^)	0.48, 0.60

Computer programs: *PAL ADSC Quantum-210 ADX* (Arvai Nielsen, 1983 ▸), *HKL3000sm* (Otwinowski Minor, 1997 ▸), *SHELXT2014* and *SHELXL2014* (Sheldrick, 2008 ▸), *DIAMOND* (Brandenburg, 2007 ▸), *WinGX* (Farrugia, 2012 ▸) and *publCIF* (Westrip, 2010 ▸).

## supplementary crystallographic information

### Crystal data

**Table:**

[Cr(NCS)_2_(C_2_H_8_N_2_)_2_]_2_[ZnCl_4_]	*Z* = 2
*M**_r_* = 783.90	*F*(000) = 796
Triclinic, *P*1	*D*_x_ = 1.683 Mg m^−^^3^
*a* = 7.6870 (15) Å	Synchrotron radiation, λ = 0.62998 Å
*b* = 13.853 (3) Å	Cell parameters from 94806 reflections
*c* = 14.560 (3) Å	θ = 0.4–33.6°
α = 92.74 (3)°	µ = 1.50 mm^−^^1^
β = 92.76 (3)°	*T* = 100 K
γ = 90.21 (3)°	Needle, red
*V* = 1546.9 (5) Å^3^	0.10 × 0.03 × 0.03 mm

### Data collection

**Table:**

ADSC Q210 CCD area-detector diffractometer	8434 reflections with *I* > 2σ(*I*)
Radiation source: PLSII 2D bending magnet	*R*_int_ = 0.014
ω scans	θ_max_ = 26.0°, θ_min_ = 2.4°
Absorption correction: empirical (using intensity measurements) (*HKL3000sm *SCALEPACK**; Otwinowski & Minor, 1997)	*h* = −10→10
*T*_min_ = 0.865, *T*_max_ = 0.956	*k* = −19→19
17036 measured reflections	*l* = −20→20
8546 independent reflections	

### Refinement

**Table:**

Refinement on *F*^2^	0 restraints
Least-squares matrix: full	Hydrogen site location: inferred from neighbouring sites
*R*[*F*^2^ > 2σ(*F*^2^)] = 0.018	H-atom parameters constrained
*wR*(*F*^2^) = 0.049	*w* = 1/[σ^2^(*F*_o_^2^) + (0.027*P*)^2^ + 0.6367*P*] where *P* = (*F*_o_^2^ + 2*F*_c_^2^)/3
*S* = 1.03	(Δ/σ)_max_ = 0.001
8546 reflections	Δρ_max_ = 0.48 e Å^−^^3^
322 parameters	Δρ_min_ = −0.60 e Å^−^^3^

### Special details

**Table:**

Geometry. All e.s.d.'s (except the e.s.d. in the dihedral angle between two l.s. planes) are estimated using the full covariance matrix. The cell e.s.d.'s are taken into account individually in the estimation of e.s.d.'s in distances, angles and torsion angles; correlations between e.s.d.'s in cell parameters are only used when they are defined by crystal symmetry. An approximate (isotropic) treatment of cell e.s.d.'s is used for estimating e.s.d.'s involving l.s. planes.

### Fractional atomic coordinates and isotropic or equivalent isotropic displacement parameters (Å^2^)

**Table:**

	*x*	*y*	*z*	*U*_iso_*/*U*_eq_	
Cr1A	1.0000	0.0000	0.5000	0.00585 (4)	
S1A	1.34965 (3)	0.21259 (2)	0.69613 (2)	0.01282 (5)	
N1A	0.83989 (11)	0.00710 (6)	0.61105 (6)	0.01133 (14)	
H1A1	0.7841	−0.0505	0.6151	0.014*	
H1A2	0.9052	0.0192	0.6642	0.014*	
N2A	0.86680 (11)	0.12458 (6)	0.46494 (6)	0.01130 (14)	
H2A1	0.9409	0.1676	0.4417	0.014*	
H2A2	0.7803	0.1099	0.4216	0.014*	
N3A	1.17486 (11)	0.08052 (6)	0.57360 (6)	0.01110 (14)	
C1A	0.70956 (13)	0.08539 (8)	0.59868 (7)	0.01647 (18)	
H1A3	0.6706	0.1094	0.6594	0.020*	
H1A4	0.6066	0.0600	0.5616	0.020*	
C2A	0.79203 (13)	0.16675 (7)	0.55045 (7)	0.01477 (18)	
H2A3	0.7036	0.2159	0.5349	0.018*	
H2A4	0.8849	0.1982	0.5909	0.018*	
C3A	1.24862 (12)	0.13654 (6)	0.62427 (6)	0.00895 (15)	
Cr2B	0.5000	0.5000	0.5000	0.00494 (4)	
S1B	0.15226 (4)	0.64435 (2)	0.26918 (2)	0.01492 (5)	
N1B	0.33365 (10)	0.38801 (6)	0.52696 (5)	0.00894 (13)	
H1B1	0.2819	0.3630	0.4735	0.011*	
H1B2	0.3944	0.3402	0.5545	0.011*	
N2B	0.38490 (10)	0.56908 (6)	0.61174 (5)	0.00906 (13)	
H2B1	0.4679	0.5980	0.6505	0.011*	
H2B2	0.3100	0.6152	0.5919	0.011*	
N3B	0.32168 (11)	0.55205 (6)	0.41316 (6)	0.01046 (14)	
C1B	0.19944 (12)	0.42703 (7)	0.58878 (7)	0.01159 (16)	
H1B3	0.1421	0.3736	0.6188	0.014*	
H1B4	0.1094	0.4622	0.5529	0.014*	
C2B	0.28916 (13)	0.49470 (7)	0.66051 (6)	0.01178 (16)	
H2B3	0.2022	0.5261	0.6999	0.014*	
H2B4	0.3714	0.4584	0.7001	0.014*	
C3B	0.24999 (11)	0.58858 (6)	0.35161 (6)	0.00807 (15)	
Cr3C	0.0000	0.5000	0.0000	0.00436 (4)	
S1C	0.47203 (3)	0.29853 (2)	−0.07639 (2)	0.01296 (5)	
N1C	0.00397 (10)	0.44890 (5)	0.13158 (5)	0.00792 (13)	
H1C1	0.1118	0.4263	0.1474	0.010*	
H1C2	−0.0225	0.4973	0.1729	0.010*	
N2C	−0.12263 (11)	0.36953 (6)	−0.03474 (5)	0.00961 (14)	
H2C1	−0.2372	0.3795	−0.0499	0.012*	
H2C2	−0.0729	0.3404	−0.0842	0.012*	
N3C	0.22291 (11)	0.43406 (6)	−0.02527 (6)	0.01166 (14)	
C1C	−0.12726 (13)	0.36960 (7)	0.13185 (6)	0.01083 (16)	
H1C3	−0.2464	0.3965	0.1322	0.013*	
H1C4	−0.1076	0.3314	0.1871	0.013*	
C2C	−0.10560 (14)	0.30666 (7)	0.04542 (6)	0.01285 (17)	
H2C3	0.0102	0.2756	0.0476	0.015*	
H2C4	−0.1961	0.2554	0.0399	0.015*	
C3C	0.32768 (12)	0.37725 (7)	−0.04576 (6)	0.00935 (15)	
Cr4D	0.5000	0.0000	0.0000	0.00523 (4)	
S1D	0.95604 (3)	0.14416 (2)	−0.15730 (2)	0.01195 (5)	
N1D	0.46882 (10)	0.12154 (5)	0.08662 (5)	0.00860 (13)	
H1D1	0.4761	0.1760	0.0545	0.010*	
H1D2	0.3627	0.1200	0.1116	0.010*	
N2D	0.65239 (10)	−0.04648 (6)	0.11111 (6)	0.01038 (14)	
H2D1	0.6223	−0.1080	0.1232	0.012*	
H2D2	0.7668	−0.0458	0.0976	0.012*	
N3D	0.70324 (10)	0.06281 (6)	−0.05272 (6)	0.01025 (14)	
C1D	0.60962 (13)	0.12127 (7)	0.16052 (6)	0.01106 (16)	
H1D3	0.5810	0.1665	0.2123	0.013*	
H1D4	0.7213	0.1420	0.1364	0.013*	
C2D	0.62447 (13)	0.01935 (7)	0.19298 (6)	0.01289 (17)	
H2D3	0.7235	0.0150	0.2386	0.015*	
H2D4	0.5166	0.0009	0.2225	0.015*	
C3D	0.80965 (12)	0.09622 (6)	−0.09656 (6)	0.00862 (15)	
Zn1E	0.22955 (2)	0.24635 (2)	0.28844 (2)	0.00695 (3)	
Cl1E	0.02321 (3)	0.32263 (2)	0.37380 (2)	0.01053 (4)	
Cl2E	0.09386 (3)	0.12821 (2)	0.20032 (2)	0.01134 (4)	
Cl3E	0.43186 (3)	0.18443 (2)	0.38989 (2)	0.01078 (4)	
Cl4E	0.37370 (3)	0.34934 (2)	0.20252 (2)	0.01048 (4)	

### Atomic displacement parameters (Å^2^)

**Table:**

	*U*^11^	*U*^22^	*U*^33^	*U*^12^	*U*^13^	*U*^23^
Cr1A	0.00661 (9)	0.00475 (8)	0.00601 (8)	−0.00088 (6)	−0.00137 (6)	0.00022 (6)
S1A	0.01664 (11)	0.00822 (9)	0.01275 (10)	−0.00177 (8)	−0.00551 (8)	−0.00184 (7)
N1A	0.0113 (3)	0.0130 (4)	0.0098 (3)	−0.0010 (3)	0.0011 (3)	0.0008 (3)
N2A	0.0132 (4)	0.0082 (3)	0.0123 (3)	0.0008 (3)	−0.0029 (3)	0.0013 (3)
N3A	0.0104 (3)	0.0111 (3)	0.0116 (3)	−0.0023 (3)	−0.0021 (3)	0.0001 (3)
C1A	0.0114 (4)	0.0213 (5)	0.0167 (4)	0.0046 (4)	0.0025 (3)	−0.0014 (4)
C2A	0.0159 (4)	0.0108 (4)	0.0168 (4)	0.0051 (3)	−0.0028 (3)	−0.0038 (3)
C3A	0.0089 (4)	0.0083 (4)	0.0098 (4)	0.0012 (3)	0.0002 (3)	0.0031 (3)
Cr2B	0.00588 (8)	0.00513 (8)	0.00366 (8)	0.00158 (6)	−0.00107 (6)	−0.00018 (6)
S1B	0.02450 (12)	0.01331 (10)	0.00660 (9)	0.00362 (9)	−0.00526 (8)	0.00280 (8)
N1B	0.0101 (3)	0.0080 (3)	0.0085 (3)	0.0000 (3)	0.0000 (3)	−0.0010 (3)
N2B	0.0108 (3)	0.0089 (3)	0.0073 (3)	0.0019 (3)	0.0004 (3)	−0.0017 (2)
N3B	0.0097 (3)	0.0120 (3)	0.0096 (3)	0.0020 (3)	−0.0020 (3)	0.0010 (3)
C1B	0.0096 (4)	0.0123 (4)	0.0131 (4)	0.0000 (3)	0.0029 (3)	0.0006 (3)
C2B	0.0149 (4)	0.0130 (4)	0.0078 (4)	0.0015 (3)	0.0036 (3)	0.0005 (3)
C3B	0.0088 (4)	0.0081 (3)	0.0072 (3)	−0.0002 (3)	0.0009 (3)	−0.0011 (3)
Cr3C	0.00541 (8)	0.00389 (8)	0.00375 (8)	0.00058 (6)	−0.00073 (6)	0.00083 (6)
S1C	0.01002 (10)	0.01022 (10)	0.01841 (11)	0.00200 (7)	0.00200 (8)	−0.00320 (8)
N1C	0.0104 (3)	0.0082 (3)	0.0051 (3)	−0.0003 (3)	−0.0013 (2)	0.0009 (2)
N2C	0.0148 (4)	0.0081 (3)	0.0059 (3)	−0.0035 (3)	−0.0017 (3)	0.0012 (2)
N3C	0.0102 (3)	0.0136 (4)	0.0116 (3)	0.0033 (3)	0.0015 (3)	0.0032 (3)
C1C	0.0145 (4)	0.0110 (4)	0.0071 (4)	−0.0040 (3)	0.0001 (3)	0.0028 (3)
C2C	0.0226 (5)	0.0071 (4)	0.0088 (4)	−0.0038 (3)	−0.0014 (3)	0.0024 (3)
C3C	0.0092 (4)	0.0105 (4)	0.0085 (4)	−0.0013 (3)	−0.0004 (3)	0.0023 (3)
Cr4D	0.00413 (8)	0.00421 (8)	0.00761 (8)	0.00135 (6)	0.00197 (6)	0.00099 (6)
S1D	0.01028 (10)	0.00953 (10)	0.01695 (11)	0.00014 (7)	0.00598 (8)	0.00466 (8)
N1D	0.0086 (3)	0.0064 (3)	0.0109 (3)	0.0017 (2)	0.0022 (3)	0.0002 (3)
N2D	0.0099 (3)	0.0084 (3)	0.0129 (3)	0.0023 (3)	−0.0004 (3)	0.0025 (3)
N3D	0.0084 (3)	0.0091 (3)	0.0134 (3)	−0.0004 (3)	0.0031 (3)	−0.0002 (3)
C1D	0.0138 (4)	0.0092 (4)	0.0101 (4)	−0.0006 (3)	−0.0003 (3)	0.0002 (3)
C2D	0.0175 (4)	0.0120 (4)	0.0094 (4)	0.0001 (3)	0.0001 (3)	0.0029 (3)
C3D	0.0081 (4)	0.0063 (3)	0.0114 (4)	0.0018 (3)	−0.0001 (3)	−0.0002 (3)
Zn1E	0.00724 (5)	0.00672 (5)	0.00674 (5)	0.00094 (3)	−0.00074 (3)	−0.00012 (3)
Cl1E	0.00871 (9)	0.01205 (9)	0.01064 (9)	0.00290 (7)	0.00053 (7)	−0.00174 (7)
Cl2E	0.01069 (9)	0.00975 (9)	0.01296 (9)	−0.00072 (7)	−0.00204 (7)	−0.00320 (7)
Cl3E	0.00939 (9)	0.01256 (9)	0.01027 (9)	0.00166 (7)	−0.00291 (7)	0.00272 (7)
Cl4E	0.01084 (9)	0.01095 (9)	0.00978 (9)	−0.00068 (7)	−0.00084 (7)	0.00329 (7)

### Geometric parameters (Å, º)

**Table:**

Cr1A—N3A^i^	1.9838 (11)	Cr3C—N2C	2.0653 (10)
Cr1A—N3A	1.9838 (11)	Cr3C—N2C^iii^	2.0653 (10)
Cr1A—N1A^i^	2.0775 (10)	Cr3C—N1C^iii^	2.0727 (9)
Cr1A—N1A	2.0776 (10)	Cr3C—N1C	2.0727 (9)
Cr1A—N2A	2.0818 (10)	S1C—C3C	1.6215 (11)
Cr1A—N2A^i^	2.0818 (10)	N1C—C1C	1.4891 (12)
S1A—C3A	1.6181 (11)	N1C—H1C1	0.9100
N1A—C1A	1.4905 (14)	N1C—H1C2	0.9100
N1A—H1A1	0.9100	N2C—C2C	1.4903 (12)
N1A—H1A2	0.9100	N2C—H2C1	0.9100
N2A—C2A	1.4912 (13)	N2C—H2C2	0.9100
N2A—H2A1	0.9100	N3C—C3C	1.1672 (13)
N2A—H2A2	0.9100	C1C—C2C	1.5125 (14)
N3A—C3A	1.1704 (13)	C1C—H1C3	0.9900
C1A—C2A	1.5094 (16)	C1C—H1C4	0.9900
C1A—H1A3	0.9900	C2C—H2C3	0.9900
C1A—H1A4	0.9900	C2C—H2C4	0.9900
C2A—H2A3	0.9900	Cr4D—N3D^iv^	1.9890 (10)
C2A—H2A4	0.9900	Cr4D—N3D	1.9890 (10)
Cr2B—N3B	1.9811 (10)	Cr4D—N1D^iv^	2.0765 (10)
Cr2B—N3B^ii^	1.9811 (10)	Cr4D—N1D	2.0766 (10)
Cr2B—N1B^ii^	2.0707 (10)	Cr4D—N2D	2.0799 (10)
Cr2B—N1B	2.0708 (10)	Cr4D—N2D^iv^	2.0799 (10)
Cr2B—N2B	2.0837 (10)	S1D—C3D	1.6237 (11)
Cr2B—N2B^ii^	2.0837 (10)	N1D—C1D	1.4891 (13)
S1B—C3B	1.6148 (10)	N1D—H1D1	0.9100
N1B—C1B	1.4879 (13)	N1D—H1D2	0.9100
N1B—H1B1	0.9100	N2D—C2D	1.4903 (13)
N1B—H1B2	0.9100	N2D—H2D1	0.9100
N2B—C2B	1.4907 (13)	N2D—H2D2	0.9100
N2B—H2B1	0.9100	N3D—C3D	1.1690 (13)
N2B—H2B2	0.9100	C1D—C2D	1.5131 (13)
N3B—C3B	1.1665 (13)	C1D—H1D3	0.9900
C1B—C2B	1.5092 (14)	C1D—H1D4	0.9900
C1B—H1B3	0.9900	C2D—H2D3	0.9900
C1B—H1B4	0.9900	C2D—H2D4	0.9900
C2B—H2B3	0.9900	Zn1E—Cl2E	2.2518 (8)
C2B—H2B4	0.9900	Zn1E—Cl4E	2.2630 (7)
Cr3C—N3C	1.9864 (10)	Zn1E—Cl3E	2.2903 (8)
Cr3C—N3C^iii^	1.9864 (10)	Zn1E—Cl1E	2.2923 (8)
			
N3A^i^—Cr1A—N3A	180.0	N3C—Cr3C—N2C^iii^	92.81 (4)
N3A^i^—Cr1A—N1A^i^	89.16 (4)	N3C^iii^—Cr3C—N2C^iii^	87.19 (4)
N3A—Cr1A—N1A^i^	90.84 (4)	N2C—Cr3C—N2C^iii^	180.0
N3A^i^—Cr1A—N1A	90.84 (4)	N3C—Cr3C—N1C^iii^	88.78 (4)
N3A—Cr1A—N1A	89.16 (4)	N3C^iii^—Cr3C—N1C^iii^	91.22 (4)
N1A^i^—Cr1A—N1A	180.0	N2C—Cr3C—N1C^iii^	96.91 (4)
N3A^i^—Cr1A—N2A	90.31 (4)	N2C^iii^—Cr3C—N1C^iii^	83.09 (4)
N3A—Cr1A—N2A	89.69 (4)	N3C—Cr3C—N1C	91.22 (4)
N1A^i^—Cr1A—N2A	97.06 (4)	N3C^iii^—Cr3C—N1C	88.78 (4)
N1A—Cr1A—N2A	82.94 (4)	N2C—Cr3C—N1C	83.09 (4)
N3A^i^—Cr1A—N2A^i^	89.69 (4)	N2C^iii^—Cr3C—N1C	96.91 (4)
N3A—Cr1A—N2A^i^	90.31 (4)	N1C^iii^—Cr3C—N1C	180.0
N1A^i^—Cr1A—N2A^i^	82.94 (4)	C1C—N1C—Cr3C	107.84 (6)
N1A—Cr1A—N2A^i^	97.06 (4)	C1C—N1C—H1C1	110.1
N2A—Cr1A—N2A^i^	180.0	Cr3C—N1C—H1C1	110.1
C1A—N1A—Cr1A	109.59 (6)	C1C—N1C—H1C2	110.1
C1A—N1A—H1A1	109.8	Cr3C—N1C—H1C2	110.1
Cr1A—N1A—H1A1	109.8	H1C1—N1C—H1C2	108.5
C1A—N1A—H1A2	109.8	C2C—N2C—Cr3C	108.84 (6)
Cr1A—N1A—H1A2	109.8	C2C—N2C—H2C1	109.9
H1A1—N1A—H1A2	108.2	Cr3C—N2C—H2C1	109.9
C2A—N2A—Cr1A	107.35 (6)	C2C—N2C—H2C2	109.9
C2A—N2A—H2A1	110.2	Cr3C—N2C—H2C2	109.9
Cr1A—N2A—H2A1	110.2	H2C1—N2C—H2C2	108.3
C2A—N2A—H2A2	110.2	C3C—N3C—Cr3C	163.96 (8)
Cr1A—N2A—H2A2	110.2	N1C—C1C—C2C	106.92 (8)
H2A1—N2A—H2A2	108.5	N1C—C1C—H1C3	110.3
C3A—N3A—Cr1A	166.35 (8)	C2C—C1C—H1C3	110.3
N1A—C1A—C2A	109.01 (8)	N1C—C1C—H1C4	110.3
N1A—C1A—H1A3	109.9	C2C—C1C—H1C4	110.3
C2A—C1A—H1A3	109.9	H1C3—C1C—H1C4	108.6
N1A—C1A—H1A4	109.9	N2C—C2C—C1C	107.87 (7)
C2A—C1A—H1A4	109.9	N2C—C2C—H2C3	110.1
H1A3—C1A—H1A4	108.3	C1C—C2C—H2C3	110.1
N2A—C2A—C1A	107.69 (8)	N2C—C2C—H2C4	110.1
N2A—C2A—H2A3	110.2	C1C—C2C—H2C4	110.1
C1A—C2A—H2A3	110.2	H2C3—C2C—H2C4	108.4
N2A—C2A—H2A4	110.2	N3C—C3C—S1C	178.85 (9)
C1A—C2A—H2A4	110.2	N3D^iv^—Cr4D—N3D	180.0
H2A3—C2A—H2A4	108.5	N3D^iv^—Cr4D—N1D^iv^	89.74 (4)
N3A—C3A—S1A	178.78 (9)	N3D—Cr4D—N1D^iv^	90.26 (4)
N3B—Cr2B—N3B^ii^	180.0	N3D^iv^—Cr4D—N1D	90.26 (4)
N3B—Cr2B—N1B^ii^	89.64 (4)	N3D—Cr4D—N1D	89.74 (4)
N3B^ii^—Cr2B—N1B^ii^	90.36 (4)	N1D^iv^—Cr4D—N1D	180.00 (3)
N3B—Cr2B—N1B	90.36 (4)	N3D^iv^—Cr4D—N2D	88.05 (4)
N3B^ii^—Cr2B—N1B	89.64 (4)	N3D—Cr4D—N2D	91.95 (4)
N1B^ii^—Cr2B—N1B	180.0	N1D^iv^—Cr4D—N2D	96.97 (4)
N3B—Cr2B—N2B	91.27 (4)	N1D—Cr4D—N2D	83.03 (4)
N3B^ii^—Cr2B—N2B	88.73 (4)	N3D^iv^—Cr4D—N2D^iv^	91.95 (4)
N1B^ii^—Cr2B—N2B	96.72 (4)	N3D—Cr4D—N2D^iv^	88.05 (4)
N1B—Cr2B—N2B	83.28 (4)	N1D^iv^—Cr4D—N2D^iv^	83.03 (4)
N3B—Cr2B—N2B^ii^	88.73 (4)	N1D—Cr4D—N2D^iv^	96.97 (4)
N3B^ii^—Cr2B—N2B^ii^	91.27 (4)	N2D—Cr4D—N2D^iv^	180.0
N1B^ii^—Cr2B—N2B^ii^	83.28 (4)	C1D—N1D—Cr4D	107.80 (6)
N1B—Cr2B—N2B^ii^	96.72 (4)	C1D—N1D—H1D1	110.1
N2B—Cr2B—N2B^ii^	180.0	Cr4D—N1D—H1D1	110.1
C1B—N1B—Cr2B	108.20 (6)	C1D—N1D—H1D2	110.1
C1B—N1B—H1B1	110.1	Cr4D—N1D—H1D2	110.1
Cr2B—N1B—H1B1	110.1	H1D1—N1D—H1D2	108.5
C1B—N1B—H1B2	110.1	C2D—N2D—Cr4D	108.84 (6)
Cr2B—N1B—H1B2	110.1	C2D—N2D—H2D1	109.9
H1B1—N1B—H1B2	108.4	Cr4D—N2D—H2D1	109.9
C2B—N2B—Cr2B	107.91 (6)	C2D—N2D—H2D2	109.9
C2B—N2B—H2B1	110.1	Cr4D—N2D—H2D2	109.9
Cr2B—N2B—H2B1	110.1	H2D1—N2D—H2D2	108.3
C2B—N2B—H2B2	110.1	C3D—N3D—Cr4D	169.62 (8)
Cr2B—N2B—H2B2	110.1	N1D—C1D—C2D	107.70 (8)
H2B1—N2B—H2B2	108.4	N1D—C1D—H1D3	110.2
C3B—N3B—Cr2B	164.42 (8)	C2D—C1D—H1D3	110.2
N1B—C1B—C2B	107.95 (8)	N1D—C1D—H1D4	110.2
N1B—C1B—H1B3	110.1	C2D—C1D—H1D4	110.2
C2B—C1B—H1B3	110.1	H1D3—C1D—H1D4	108.5
N1B—C1B—H1B4	110.1	N2D—C2D—C1D	107.87 (8)
C2B—C1B—H1B4	110.1	N2D—C2D—H2D3	110.1
H1B3—C1B—H1B4	108.4	C1D—C2D—H2D3	110.1
N2B—C2B—C1B	107.96 (7)	N2D—C2D—H2D4	110.1
N2B—C2B—H2B3	110.1	C1D—C2D—H2D4	110.1
C1B—C2B—H2B3	110.1	H2D3—C2D—H2D4	108.4
N2B—C2B—H2B4	110.1	N3D—C3D—S1D	179.15 (9)
C1B—C2B—H2B4	110.1	Cl2E—Zn1E—Cl4E	111.63 (2)
H2B3—C2B—H2B4	108.4	Cl2E—Zn1E—Cl3E	111.31 (2)
N3B—C3B—S1B	177.11 (8)	Cl4E—Zn1E—Cl3E	106.71 (2)
N3C—Cr3C—N3C^iii^	180.0	Cl2E—Zn1E—Cl1E	107.46 (2)
N3C—Cr3C—N2C	87.19 (4)	Cl4E—Zn1E—Cl1E	112.49 (2)
N3C^iii^—Cr3C—N2C	92.81 (4)	Cl3E—Zn1E—Cl1E	107.20 (2)
			
Cr1A—N1A—C1A—C2A	−33.94 (9)	Cr3C—N1C—C1C—C2C	44.29 (8)
Cr1A—N2A—C2A—C1A	−45.42 (9)	Cr3C—N2C—C2C—C1C	39.21 (9)
N1A—C1A—C2A—N2A	52.98 (10)	N1C—C1C—C2C—N2C	−55.56 (10)
Cr2B—N1B—C1B—C2B	−41.73 (8)	Cr4D—N1D—C1D—C2D	−43.80 (8)
Cr2B—N2B—C2B—C1B	−40.80 (8)	Cr4D—N2D—C2D—C1D	−38.66 (9)
N1B—C1B—C2B—N2B	55.28 (10)	N1D—C1D—C2D—N2D	55.05 (10)

Symmetry codes: (i) −*x*+2, −*y*, −*z*+1; (ii) −*x*+1, −*y*+1, −*z*+1; (iii) −*x*, −*y*+1, −*z*; (iv) −*x*+1, −*y*, −*z*.

### Hydrogen-bond geometry (Å, º)

**Table:**

*D*—H···*A*	*D*—H	H···*A*	*D*···*A*	*D*—H···*A*
N1*A*—H1*A*1···Cl3*E*^v^	0.91	2.48	3.3700 (13)	165
N2*A*—H2*A*1···Cl1*E*^vi^	0.91	2.50	3.3483 (13)	155
N2*A*—H2*A*2···Cl3*E*	0.91	2.90	3.5797 (12)	133
C1*A*—H1*A*3···S1*A*^vii^	0.99	2.91	3.5983 (15)	127
C2*A*—H2*A*3···Cl3*E*	0.99	2.91	3.5533 (15)	123
C2*A*—H2*A*4···S1*B*^ii^	0.99	2.94	3.6270 (15)	128
N1*B*—H1*B*1···Cl1*E*	0.91	2.45	3.2813 (13)	152
N1*B*—H1*B*2···S1*A*^vii^	0.91	2.81	3.5401 (13)	138
N2*B*—H2*B*1···Cl4*E*^ii^	0.91	2.49	3.3532 (13)	159
N2*B*—H2*B*2···Cl1*E*^viii^	0.91	2.77	3.4934 (12)	138
C1*B*—H1*B*3···S1*A*^vii^	0.99	2.98	3.5910 (14)	121
C1*B*—H1*B*3···S1*B*^viii^	0.99	2.87	3.6440 (14)	136
C2*B*—H2*B*3···Cl1*E*^viii^	0.99	2.93	3.5309 (14)	120
N1*C*—H1*C*1···Cl4*E*	0.91	2.40	3.3058 (12)	171
N1*C*—H1*C*2···S1*B*	0.91	2.73	3.4473 (14)	137
N2*C*—H2*C*1···S1*C*^vii^	0.91	2.50	3.2836 (12)	144
N2*C*—H2*C*2···S1*B*^iii^	0.91	2.75	3.4063 (11)	130
N2*C*—H2*C*2···S1*D*^vii^	0.91	2.88	3.5893 (13)	135
C1*C*—H1*C*4···Cl1*E*	0.99	2.86	3.7421 (13)	149
N1*D*—H1*D*1···S1*C*	0.91	2.61	3.4937 (13)	164
N1*D*—H1*D*2···Cl2*E*	0.91	2.49	3.3919 (12)	172
N2*D*—H2*D*1···S1*C*^iv^	0.91	2.78	3.6225 (12)	155
N2*D*—H2*D*2···S1*D*^ix^	0.91	2.67	3.3564 (12)	133
C1*D*—H1*D*3···Cl3*E*	0.99	2.88	3.7357 (14)	145
C1*D*—H1*D*4···Cl2*E*^vi^	0.99	2.98	3.7397 (12)	135

Symmetry codes: (ii) −*x*+1, −*y*+1, −*z*+1; (iii) −*x*, −*y*+1, −*z*; (iv) −*x*+1, −*y*, −*z*; (v) −*x*+1, −*y*, −*z*+1; (vi) *x*+1, *y*, *z*; (vii) *x*−1, *y*, *z*; (viii) −*x*, −*y*+1, −*z*+1; (ix) −*x*+2, −*y*, −*z*.
